# Identification of PDGFRα-positive interstitial cells in the distal segment of the murine vas deferens

**DOI:** 10.1038/s41598-021-87049-6

**Published:** 2021-04-06

**Authors:** Tasuku Hiroshige, Kei-Ichiro Uemura, Shingo Hirashima, Kiyosato Hino, Akinobu Togo, Keisuke Ohta, Tsukasa Igawa, Kei-Ichiro Nakamura

**Affiliations:** 1 Division of Microscopic and Development Anatomy, Department of Anatomy, Kurume University School of Medicine, Kurume, 830-0011 Japan; 2 Department of Urology, Kurume University School of Medicine, Kurume, 830-0011 Japan; 3 Advanced Imaging Research Center, Kurume University School of Medicine, Kurume, 830-0011 Japan

**Keywords:** Cellular imaging, Anatomy, Urology

## Abstract

Platelet-derived growth factor receptor-α (PDGFRα)-positive interstitial cells (ICs) are widely distributed in various organs and may be involved in the motility of various tubular organs. We, for the first time, aimed to investigate the distribution, immunohistochemical characteristics, and ultrastructure of PDGFRα-positive ICs in murine vas deferens, using confocal laser scanning microscopy, transmission electron microscopy (TEM), and immuno-electron microscopy (immuno-EM). For immunofluorescence, we used antibodies against PDGFRα and other markers of ICs. PDGFRα-positive ICs were distributed widely in the lamina propria, smooth muscles, and serosal layers. Although most PDGFRα-positive ICs labeled CD34, they did not label CD34 in the subepithelial layers. Additionally, PDGFRα-positive ICs were in close proximity to each other, as also to the surrounding cells. TEM and immuno-EM findings revealed that PDGFRα-positive ICs established close physical interactions with adjacent ICs. Extracellular vesicles were also detected around the PDGFRα-positive ICs. Our morphological findings suggest that PDGFRα-positive ICs may have several subpopulations, which can play an important role in intercellular signaling via direct contact with the IC network and the extracellular vesicles in the murine vas deferens. Further investigation on PDGFRα-positive ICs in the vas deferens may lead to understanding the vas deferens mortility.

## Introduction

The vas deferens plays an important role in male ejaculation, during which the smooth muscle in the wall contract reflexively, propelling the sperm forward. This is also known as peristalsis^1^. Peristalsis of the vas deferens transports sperm from the epididymis to the ampulla, from which the seminal vesicle secretions of the sperm are propelled forward through the ejaculatory ducts toward the urethra, using synchronized rhythmic contractions of the smooth muscle. Therefore, the vas deferens plays a crucial role in transporting sperm in the genital organs^2^, and understanding vas deferens motility is important to revealing the mechanism of sperm transport.

In the case of gastrointestinal tract motility, which is the same smooth muscle organs as vas deferens, interstitial cells of Cajal (ICC) act as pacemakers for peristaltic movement. The ICC are c-Kit immunoreactive and have been extensively studied previously^3–6^. They generate spontaneous electrical depolarizations that organize contractile activity into patterns of phasic contractions and facilitate a pathway for active transmission of electrical activity in smooth muscles^5^. ICC can be easily identified by their unique ultrastructural feature, since they have small cell bodies and several elongated processes whose length reaches approximately 100 μm. They also have numerous mitochondria and caveolae, abundant intermediate filaments, a discontinuous basal lamina, moderately developed Golgi apparatus, rough and smooth endoplasmic reticula, close contact with nerve varicosities and formation of numerous gap junctions with each other and with smooth muscle cells^7^.

Recently, platelet-derived growth factor receptor-α (PDGFRα)-positive interstitial cells (ICs), with ultrastructural characteristics similar to those of ICC, have been reported in various organs other than the gastrointestinal tract^8^. These ICs mostly do not express c-Kit. Like c-Kit, PDGFRα is a tyrosine-protein kinase that acts as a cell-surface receptor for certain isoforms of platelet-derived growth factors (PDGFs), and stimulates cell signaling pathways that elicit responses such as cellular growth and differentiation^9^. The most common theory is that they participate in intercellular signaling and may be responsible for the integration of signals from numerous systems such as nervous, vascular and immune systems. In smooth muscle organs, they have been suggested to regulate smooth muscle motility^10,11^, as reported by physiological functional studies on human and murine colon, murine renal pelvis, and murine bladder^12–15^. Additionally, PDGFRα-positive ICs enable the maintenance and proper organization of the extracellular matrix and facilitates cell migration during organogenesis^16^. Therefore, they have a potential role in the etiopathogenesis of different diseases^17^. Although PDGFRα-positive ICs in the male genitalia have been reported in the testes^18,19^ and seminal vesicles^20^, they have not been investigated in the vas deferens.

Here, we aimed to identify PDGFRα-positive ICs in murine vas deferens, and characterize their morphological features, ultrastructure, and positional relationship with other tissues (smooth muscle bundles, nerve tracts, blood vessels, epithelial cells, and macrophages) using immunohistochemistry, transmission electron microscopy (TEM), and immune-electron microscopy (immune-EM) for the first time. Our results will help in understanding the potential function of PDGFRα-positive ICs and their involvement in vas deferens motility.

## Methods

### Animals

Three green fluorescent protein (GFP) transgenic male mice (C57BL/6-Tg (CAG-EGFP)1Osb/J)^21^ and three wild-type (WT) male mice (C57BL/6) were used in this study. All the mice were bred at the institution's animal facility. GFP transgenic mice and wild-type mice were sacrificed by the method described below at the age of 3–6 months. All mice were fed ad libitum and bred under a normal 12-h light/dark schedule. All the experiments were performed in accordance with the National Institutes of Health Guidelines for Animal Research and ARRIVE (Animal Research: Reporting of In Vivo Experiments) guidelines (https://www.nc3rs.org.uk/arrive-guidelines). All the animal procedures were approved by the Board for Animal Experiments of Kurume University.

### Tissue clearing using the clear unobstructed brain imaging cocktails and computational analysis (CUBIC) protocol and multi-photon deep imaging

GFP transgenic male mice were anesthetized with sodium pentobarbital (50 mg/kg). The mice were then fixed in a supine position with their necks extended and were then transcardially perfused through the left ventricle with heparin containing (10 U/mL) saline, followed by fixation with 4% paraformaldehyde in phosphate buffered saline (PBS). After perfusion, a median incision was made to open the abdominal cavity to harvest bilateral pars vas deferens. The dissected specimens were immersed in the same fixative overnight at 4 °C. After washing with PBS, the post-fixed specimens were immersed in 50% CUBIC-L solution (T3740, Tokyo Chemical Industry) in distilled water on a shaker at 37 °C overnight. Next, the specimens were immersed in 100% CUBIC-L solution on a shaker at 37 °C for 5 days and 100% CUBIC-L solution was refreshed every other day. Following delipidation, the specimens were washed in PBS overnight at room temperature (22–25 °C). The specimens were then immersed in 50% CUBIC-R^+^ solution (T3740, Tokyo Chemical Industry) in distilled water on a shaker at room temperature (22–25 °C) overnight, and then immersed in CUBIC-R^+^ solution on a shaker at room temperature for an additional 1 or 2 days and later embedded in CUBIC-R^+^. The transparent specimens were observed under a multi-photon microscope (FVMPE-RS, Olympus, Japan) with acquisition parameters as follows: Excitation at 1000 nm, a 10 × 0.6NA SCALEVIEW-A2 immersion lens (XLPLN10XSVMP, Olympus, Japan) and an image size of 1271 × 1272 μm. Image deconvolution and three-dimensional (3D) reconstruction were performed with the resulting image stacks and was analyzed using Avizo software (version 9.1.1, FEI, USA) available at https://www.fei.com/software/avizo3d/.

### Hematoxylin and Eosin (H&E) staining

Wild type male mice were deeply anesthetized and transcardially perfused with 4% PFA in PBS as previously described. Dissected specimens were immersed in the same fixative for 2 h at 4 °C. The specimens were subsequently trimmed, washed three times for 5 min in PBS, and then immersed in PBS containing 30% sucrose overnight at 4 °C, after which they were frozen in optical cutting temperature (O.C.T.) compound (Tissue-Tek, Sakura Finetek, USA). Frozen blocks were cut into 4-μm thick sections using a cryomicrotome (CM1950, Leica, Germany). The sections were stained with H&E and subsequently imaged.

### Immunofluorescence histochemistry and confocal laser scanning microscope (CLSM) imaging

Aforementioned cryosections (4 μm) were placed on slide glasses (refer to the text of H&E staining). The sections were incubated with either 3% normal goat serum or 3% normal donkey serum and 0.5% Triton X-100 in PBS for 30 min to block non-specific binding, and then incubated with primary antibodies diluted in blocking solution overnight at 4 °C (see Supplementary Table S1 online). The sections were then incubated with secondary antibodies for 1 h (1:1000 dilution) at room temperature (22–25 °C). Alexa Fluor-488-conjugated donkey anti-goat IgG, Alexa Fluor-488-conjugated goat anti-rat IgG, Alexa Fluor-488-conjugated goat anti-chicken IgG, Alexa Fluor-568-conjugated donkey anti-rat IgG, Alexa Fluor-647-conjugated donkey anti-chicken IgG, Alexa Fluor-568-conjugated donkey anti-rabbit IgG (all 1:500 dilution; Invitrogen, USA) were used as secondary antibodies. After washes with PBS, the immunolabeled sections were mounted using PermaFluor aqueous mounting medium (PermaFluor, Thermo Scientific, USA) and were observed under a CLSM (FV1000, Olympus, Japan) with following acquisition parameters: excitation at 473 and 559 nm, × 60 water immersion lens (NA = 1.2), image size = 105 × 105 μm.

### Transmission electron microscopy (TEM)

Wild type male mice were deeply anesthetized and transcardially perfused with half *Karnovsky* fixative (2% paraformaldehyde, 2.5% glutaraldehyde, 2 mM CaCl_2_ in 0.1 M cacodylate buffer [pH 7.3]) as previously described. Dissected specimens, cut into 1-mm cubes, were immersed in the same fixative for 2 h at 4 °C as a post-fixation and were washed in 0.1 M cacodylate buffer three times for 10 min each. Next, the specimens were prepared according to the *en bloc* stain procedure as follows: the specimens were post-fixed for 2 h in a solution containing 2% osmium tetraoxide and 1.5% potassium ferrocyanide in 0.2 M cacodylate buffer at 4 °C, and were then washed three times with distilled water. They were then treated in 1% aqueous thiocarbohydrazide solution for 1 h, and immersed in a solution containing 2% osmium tetroxide in distilled water after five washes with distilled water. Later, they were washed three times with distilled water. The specimens were immersed in 4% uranyl acetate in distilled water overnight and were then washed five times with distilled water. The specimens were then stained using Walton’s lead aspartate solution for 1 h^22^, and dehydrated using ice-chilled ethanol gradient series (20%, 50%, 70%, 80%, 90%, and twice in 100% for 10 min each) and ice-chilled 100% acetone for 10 min. Dehydrated specimens were embedded by infiltration with epoxy resin (Epon 812, TAAB, England) mixture, and were polymerized for 72 h at 60 °C. The surfaces of the specimens were exposed by trimming, and ultrathin sections with a thickness of 60 nm were cut from embedded specimens using a diamond knife on an ultramicrotome (Ultracut E microtome, Reichert-Jung, Germany). They were placed on copper-coated grids. The ultrathin sections on the grids were observed using a TEM (H-7000, Hitachi high technology, Japan) and were photographed using a high-resolution 16 mega-pixel digital camera (Advanced Microscopy Techniques, USA) connected to the TEM.

### Post-embedding immune-electron microscopy (immuno-EM)

Wild type male mice were deeply anesthetized and sodium pentobarbital (50 mg/kg) and were transcardially perfused through the left ventricle with heparin containing (10 U/mL) saline, followed by fixation with 4% paraformaldehyde (PFA)/0.1% glutaraldehyde in 0.1 M cacodylate buffer (pH 7.3). After fixation, the specimens were rinsed with 0.1 M cacodylate buffer (pH 7.3). They were subsequently dehydrated in an ethanol series (50%, 70%, and 90% for 30 min each). Finally, the tissues were embedded in LR white and polymerized at 60 °C for 24 h. Post-embedding, immunogold labeling was performed as previously outlined by Valtschanoff et al.^19^. The surfaces of the embedded specimens were exposed, and ultrathin sections (70 nm) were cut using a diamond knife on an ultramicrotome (Ultracut E microtome, Reichert-Jung, Germany) and collected on nickel mesh grids to preserve the cutting order. After washing with PBS, the grids were blocked with 1% bovine serum albumin in 0.05 M Tris-buffered saline with 0.5% triton X-100 (TBS-T, pH 7.6), followed by overnight treatment with goat anti-PDGFRα (1:100 dilution; catalog no. AF1062; R&D systems, USA) diluted in blocking solution overnight at 4 °C. Grids were subsequently treated with a secondary antibody conjugated to gold colloids with 10 nm mean particle size (1:100 dilution; catalog no. EMRAG10; BBInternational, UK). After immunoprocessing, the sections were post-stained using uranyl acetate and Sato's lead salts. The grids were examined under a TEM (H-7000, Hitachi high technology, Japan) and photographed using a high-resolution 16 mega-pixel digital camera (Advanced Microscopy Techniques, USA) connected to the TEM.

## Results

### Multi-photon microscopic observations of the wholemount murine vas deferens using CUBIC

**Figure 1:**
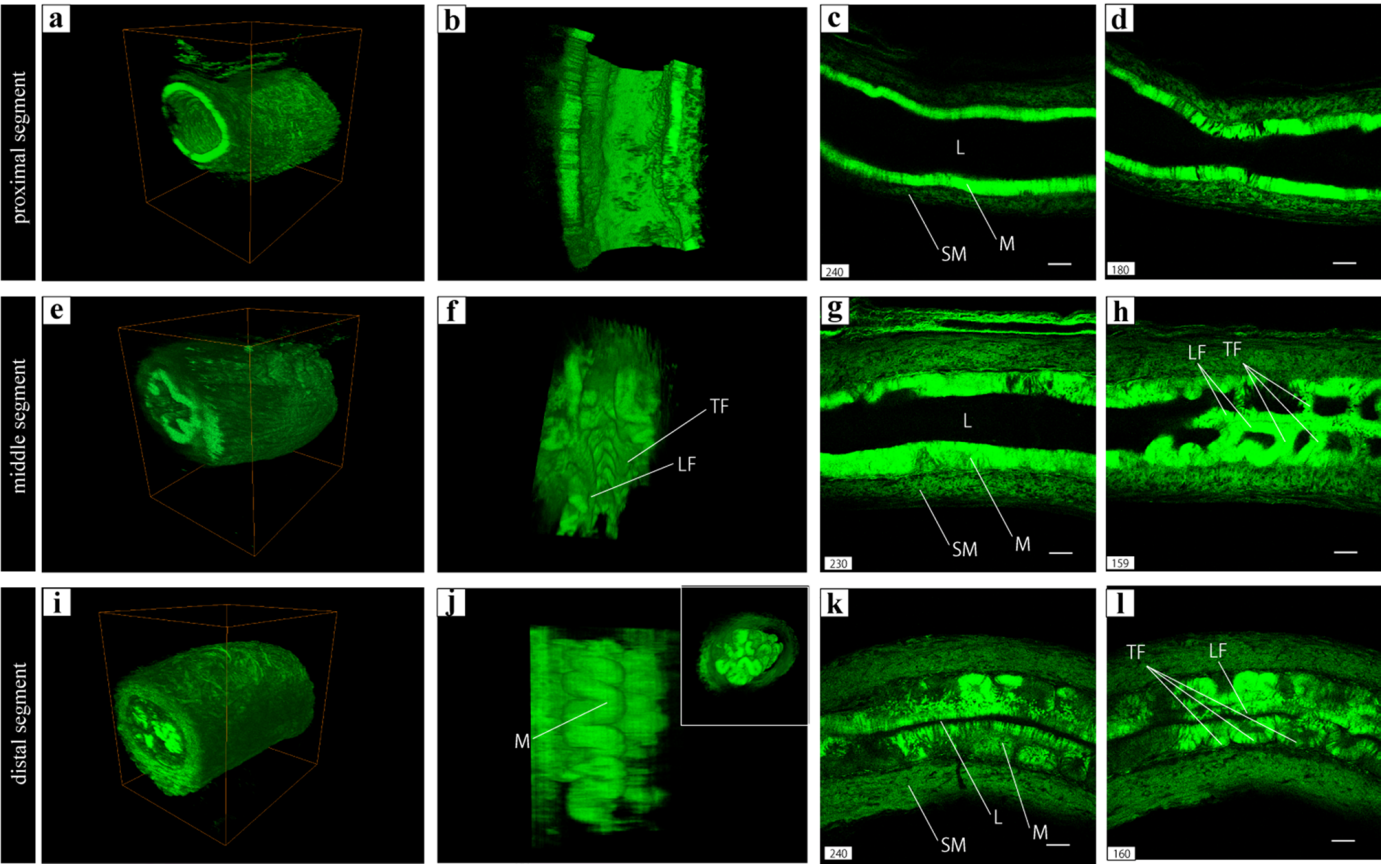
(**a**) The 3D reconstructed overview of the proximal segment of GFP transgenic murine vas deferens. (**b**) Lumens of (**a**). (**c**,**d**) Sagittal sections including the lumen of (**a**). (**e)**: 3D reconstructed overview of the middle segment of GFP transgenic murine vas deferens. (**f**) Lumens of (**e**). (**g**,**h**) Sagittal sections including the lumen of (**e**). (**i)**: 3D reconstructed overview of the distal segment of GFP transgenic murine vas deferens. (**j**): Side view of the mucosa area of (**i**). Front view of the mucosa area of (**i**) (inset at the upper right corner of (**j**)). (**k**,**l**): Sagittal sections including the lumen of (**i**). The same images following deconvolution and 3D reconstruction using the Avizo software (version 9.1.1) available at https://www.fei.com/software/avizo3d/ (**a**,**b**,**e**,**f**,**i**,**j**). *LF* longitudinal fold, *TF* transverse fold, *M* mucosa, *L* lumen, *SM* smooth muscle. Scale bars: 100 µm (**c**,**d**,**g**,**h**,**k**,**l**).

We performed tissue clearing of murine vas deferens using the CUBIC method and observed the transparent wholemount vas deferens using multi-photon microscopy (see Supplementary Fig. S1 online). The observed two-dimensional (2D) images of each segment were reconstructed in 3D (Fig. 1a,e,i). The proximal segment of murine vas deferens, which was about a quarter of the entire length, had a thin lamina propria and the lumen was highly distended (Fig. 1b–d). From the proximal segment to the central portion, longitudinal folds were observed (Fig. 1f,h), and the lumen gradually narrowed (Fig. 1g). The transverse folds appeared at equal intervals and extended straight towards one another (Fig. 1f,h). In the distal segment from the center part to the ampulla, the number of longitudinal, and transverse folds and the length of the longitudinal folds increased, and the lumen became narrower (Fig. 1k,l). The mucosa between the longitudinal folds extended like a sine curve as the transverse folds intersected with one another (Fig. 1j, right upper square). Our results showed that the distal segment, containing several folds, complex lumen, and this smooth muscle layer, was the identification target.

### H&E staining

**Figure 2:**
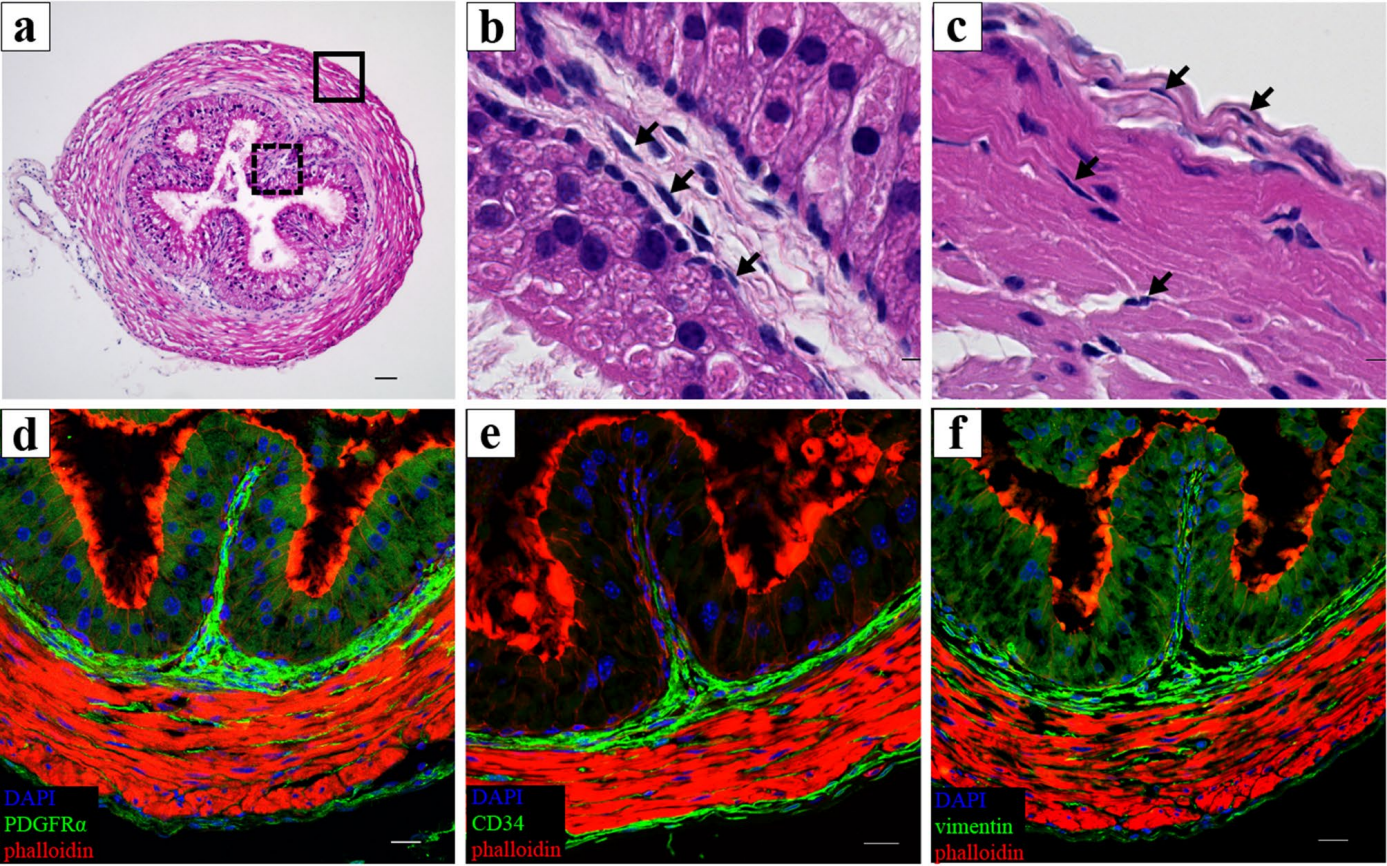
(**a**) The cross-sectional images of Hematoxylin and Eosin stained murine vas deferens. (**b**) High magnification of the solid black line square area of (**a**). Several cells with small cell bodies and elongated cellular processes were observed in the lamina propria (**b**, black arrows). (**c**) High magnification of the dotted black square area. These cells were broadly distributed in the smooth muscle and serosal layers (**c**, black arrows). (**d**) Representative image of the double immunolabeling for PDGFRα (green) and phalloidin (red). (**e**) Representative image of the double immunolabeling for CD34 (green) and phalloidin (red). (**f**) Representative image of double immunolabeling for vimentin (green) and phalloidin (red). Nuclei were counterstained in blue with 4′,6-diamidino-2-phenylindole (**d**–**f**). Immunoreactivities of all the markers observed in the lamina propria, the smooth muscle and the serosal layers (**d**–**f**). Scale bars: 50 µm (**a**,**d–f**), 5 µm (**b**,**c**).

We identified several cells that had small cell bodies and elongated cellular processes in cross-sectional images of H&E stained murine vas deferens (Fig. 2a). These cells were organized in the lamina propria (Fig. 2b, black arrows), in the smooth muscle and the serosal layers (Fig. 2c, black arrows).

### CLSM observation of immunofluorescence

Immunoreactivities of all the markers (PDGFRα, CD34, and vimentin) in labeling ICs were observed in the entire lamina propria and in between the layer of smooth muscle bundles (Fig. 2d–f). In addition, two to three layers of these immunoreactivities were observed outside the smooth muscle layer (Fig. 2d–f).

**Figure 3:**
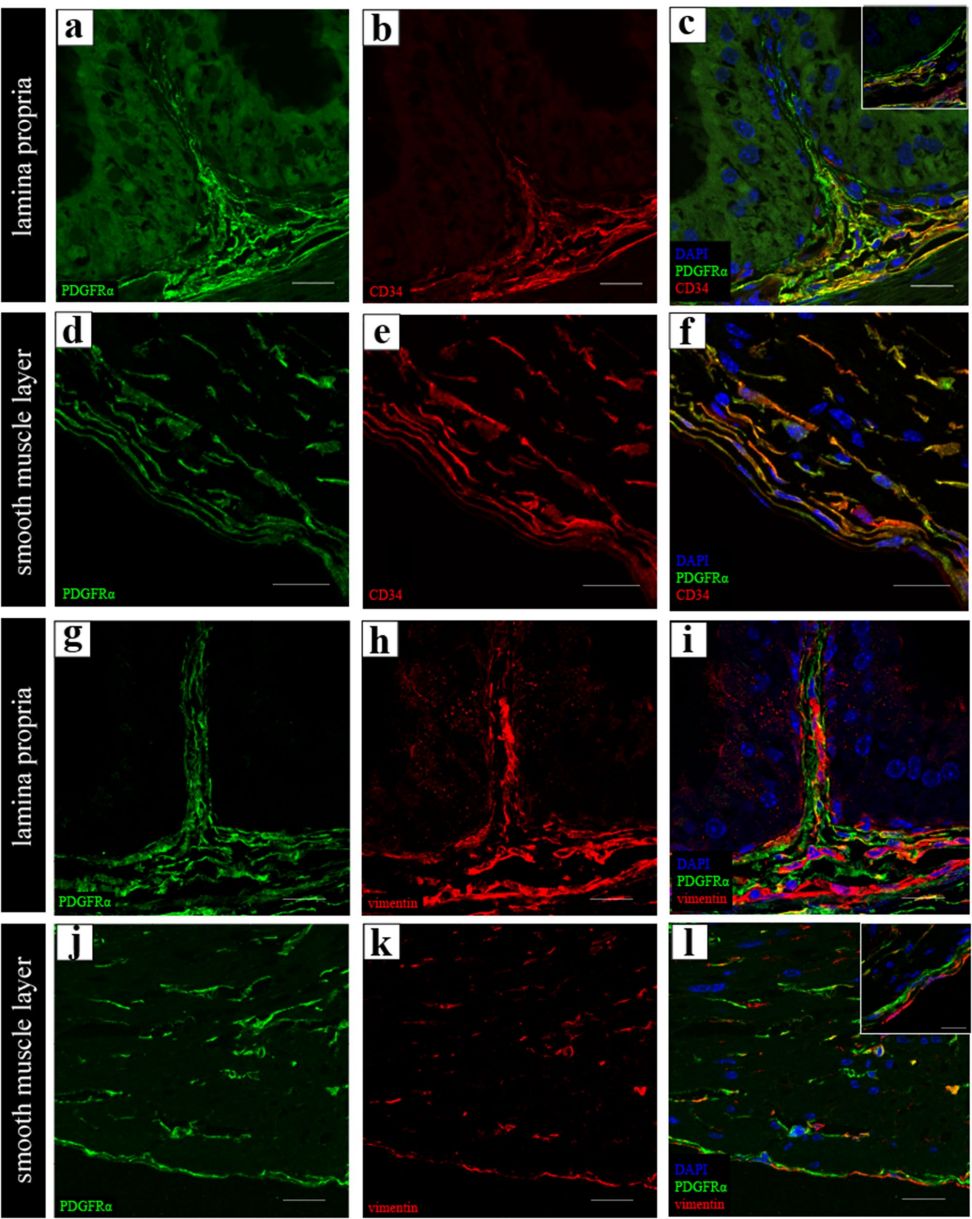
(**a–f**) Representative images of the double immunolabeling for PDGFRα (green) and CD34 (red) in the lamina propria (**a**–**c**) and in the smooth muscle layer (**d**–**f**). High magnification in the area beneath the epithelium (inset at the upper right corner of (**c**)). PDGFRα-immunoreactivity (IR) observed beneath the epithelium was not co-labeled with CD34-IR (**c**). PDGFRα-IR observed within the deeper portions of the lamina propria, in the smooth muscle and serosal layers were co-labeled with CD34 (**c**,**f**). (**g**–**l**) Representative images of the double immunolabeling for PDGFRα (green) and vimentin (red) in the lamina propria (**g**–**i**), in the smooth muscle layer and serosal layer (**j**–**l**). PDGFRα-IR was partially co-labeled with vimentin-IR in all layers (**c**,**f**). High magnification in the serosal layer (inset at the upper right corner of (**l**)). Vimentin-single IR cells were observed in the outermost of serosal layer (**f**). Nuclei were counterstained in blue with 4′,6-diamidino-2-phenylindole (**c**,**f**,**i**,**l**). Scale bars: 20 µm (**a**–**l**), 5 µm (inset at the upper right corner of (**c**,**l**)).

In double immunolabeling for PDGFRα and CD34 (Fig. 3a–f), PDGFRα-immunoreactivity (IR) observed beneath the epithelium was not co-labeled with CD34-IR (Fig. 3c, right upper square). PDGFRα-IR observed in deeper portions within the lamina propria in the smooth muscle layer and the serosal layer was co-labeled with CD34-IR (Fig. 3c,f). In double immunolabeling for PDGFRα and vimentin (Fig. 3g–l), PDGFRα-IR was partially co-labeled with vimentin-IR in all the layers (Fig. 3i,l). The PDGFRα-IR homogeneously labeled cell bodies and cell processes, whereas vimentin-IR primarily labeled the cytoplasm and partially labeled the cell processes (Fig. 3i,l). The vimentin-single IR was frequently observed in the lamina propria (Fig. 3i). In double immunolabeling for vimentin and laminin (see Supplementary Fig. S2a online), the vimentin-IR was enclosed by laminin-IR, indicating the presence of a basal membrane of vascular endothelial cells. Therefore, the vimentin-single IR was considered to be a different type of mesenchymal cells, including the vascular endothelial cells. Vimentin-single IR was also observed in the outermost serosal layer and could be considered as mesothelial cells (Fig. 3l, right upper square, see Supplementary Fig. S2b online).

**Figure 4:**
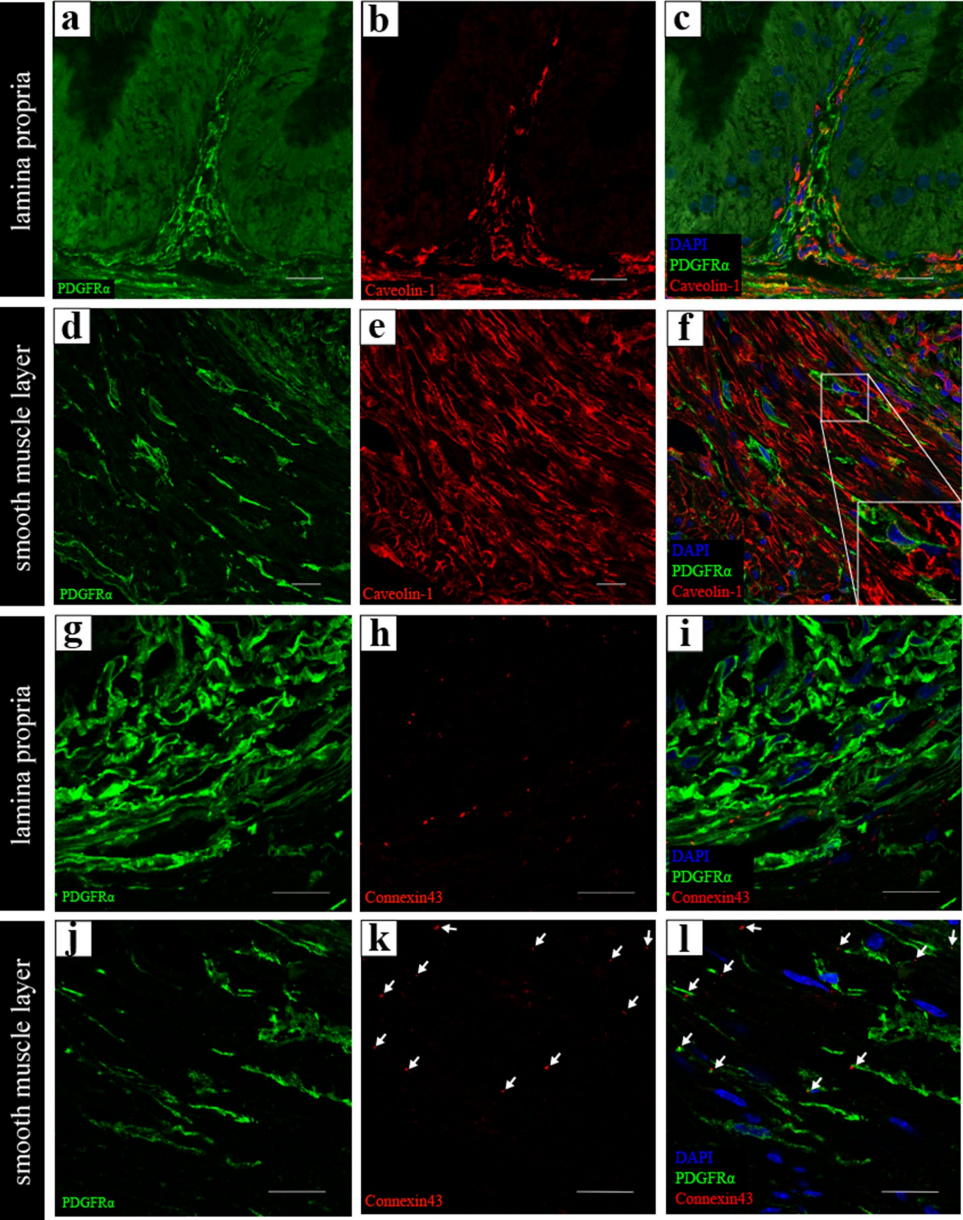
(**a**–**e**) Representative images of the double immunolabeling for PDGFRα (green) and caveolin-1 (red) in the lamina propria (**a**–**c**) and in the smooth muscle layer (**b**–**e**). PDGFRα-immunoreactivity (IR) was partially co-labeled with caveolin-1-IR in all layers (**c**,**f**). High magnification in the white line square area (inset at the lower right corner of (**f**)). (**g**–**l**) Representative images of the double immunolabeling for PDGFRα (green) and connexin-43 (red) in the lamina propria (**g**–**i**) and in the smooth muscle layer (**j**–**l**). Connexin 43-IR was observed surrounding PDGFRα-IR in the lamina propria (**i**). However, Connexin 43-IR (white arrows) was less prominent in the smooth muscle layer than in the lamina propria (**l**). Nuclei were counterstained in blue with 4′,6-diamidino-2-phenylindole (**c**,**f**,**i**,**l**). Scale bars: 20 µm (**a**–**l**), 5 µm (inset at the lower right corner of (**f**)).

Furthermore, double immunolabeling for PDGFRα and caveolin-1 shows PDGFRα-IR was partially co-labeled with caveolin-1-IR in all the layers (Fig. 4a–f). Caveolin-IR was observed heterogeneously in PDGFRα-IR (Fig. 4f, right upper square). The distribution pattern of the caveolin-1-single IR observed in the lamina propria was consistent with the localization of vascular endothelial cells, such as vimentin-single IR (Fig. 4c). The distribution pattern of caveolin-1-single IR observed in the smooth muscle layer was consistent with the localization of smooth muscle cells (see Supplementary Fig. S2c online). Double immunolabeling for PDGFRα and connexin-43 was performed to observe gap junction mediated cellular signaling network (Fig. 4g–l). Connexin 43-IR was observed surrounding PDGFRα-IR in the lamina propria (Fig. 4i). However, connexin 43-IR was less prominent in the smooth muscle layers than in the lamina propria (Fig. 4l, white arrows).

**Figure 5:**
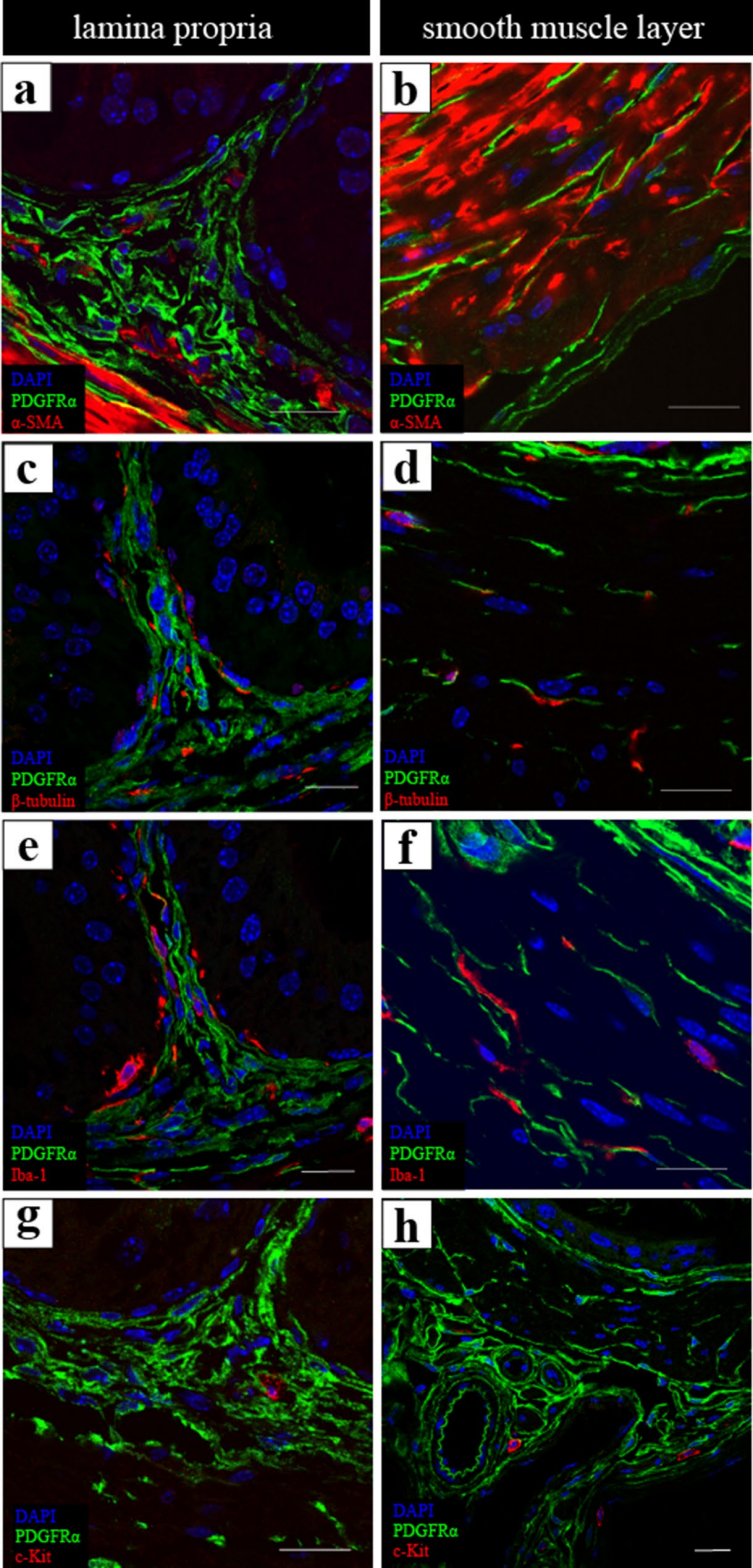
(**a**,**b**) Representative images of the double immunolabeling for PDGFRα (green) and α-smooth muscle actin (αSMA; red) in the lamina propria (**a**) and in the smooth muscle layer (**b**). PDGFRα-immunoreactivity (IR) was observed around vascular smooth muscle cells labeled with αSMA-IR in the lamina propria (**a**). Vascular smooth muscle cells were indistinguishable from smooth muscle cells in the smooth muscle layer (**b**). (**c**,**d**): Representative images of the double immunolabeling for PDGFRα (green) and β3-tubulin (red) in the lamina propria (**c**) and in the smooth muscle layer (**d**). Nerves labeled with β3-tubulin were observed in close proximity to some PDGFRα-IR cells in the lamina propria and smooth muscle layer (**c**,**d**). (**e**,**f**) Representative images of the double immunolabeling for PDGFRα (green) and ionized calcium-binding adaptor molecule 1 (Iba-1; red) in the lamina propria (**e**) and in the smooth muscle layer (**f**). Macrophages labeled with Iba-1 were observed in close proximity to some PDGFRα-IR in the lamina propria and smooth muscle layer same as nerves and blood vessels (**e**,**f**). (**g**,**h**) Representative images of the double immunolabeling for PDGFRα (green) and c-Kit (red) in the lamina propria (**g**) and in the smooth muscle layer (**h**). c-Kit-IR was not observed in all the layers of the vas deferens except for mast cells (**g**,**h**). Scale bars: 20 µm (**a**–**h**).

To observe the relationship between PDGFRα-IR and other type of cells, double immunolabeling for PDGFRα and α-smooth muscle actin (αSMA, smooth muscle cells), β3-tubulin (nerves), or ionized calcium-binding adaptor molecule 1 (Iba-1, macrophages) was performed. PDGFRα-IR was found around vascular smooth muscle cells labeled with αSMA-IR in the lamina propria (Fig. 5a). In the smooth muscle layer, vascular smooth muscle cells were indistinguishable from smooth muscle cells of the vas deferens (Fig. 5b). Nerves labeled with β3-tubulin were found in close proximity to some PDGFRα-IR in the lamina propria and smooth muscle layer (Fig. 5c,d). Macrophages labeled with Iba-1 were also found in close proximity to PDGFRα-IR in the lamina propria and smooth muscle layer (Fig. 5e,f). Finally, we attempted to perform double immunolabeling to examine whether c-Kit-IR, which labels ICC in the gastrointestinal tract, was observed. c-Kit-IR was only observed in mast cell-like cells (Fig. 5g,h). As a positive control, we also processed the tunica muscularis of the murine colon using the same protocols as the studies on vas deferens. c-Kit-IR was observed routinely in the ICC of the colon (see Supplementary Fig. S2d online). These data verified the suitability of the antibodies and techniques used in the present study to detect c-Kit-IR.

To evaluate the expression of Ca^2+^-activated K^+^ (SK3) and Ca^2+^-activated Cl^−^ (ANO-1) channels, which have been previously reported in PDGFRα-positive ICs of other smooth muscle organs^12–14^, in PDGFRα-positive ICs of the smooth muscle layer of the murine vas deferens, we double immunostained for PDGFRα and SK3, and for PDGFRα and ANO-1, and found that PDGFRα-IR was not co-labeled with SK3-IR and ANO-1-IR (data not shown).

### Ultrastructural characterization of interstitial tissue of murine vas deferens using TEM

**Figure 6:**
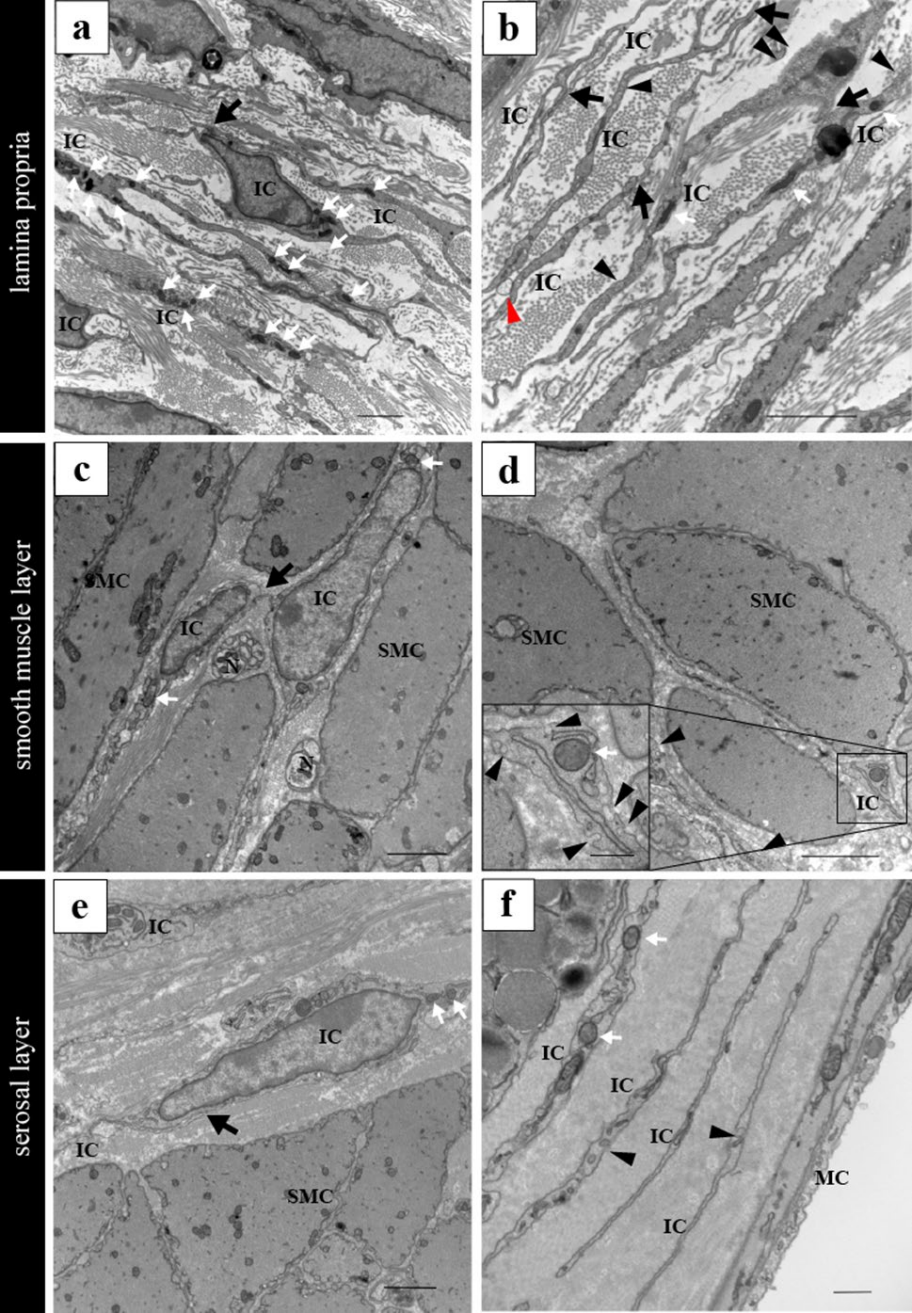
(**a**) ICs in the lamina propria of the vas deferens. (**b**) Cell processes of ICs in the lamina propria. Exosome-like structure between cell processes of ICs (red arrowhead). (**c**) ICs in the smooth muscle layer of the vas deferens. (**d**) Cell processes of ICs in the smooth muscle layer. High magnification of the solid black line square area (inset at the lower left corner of (**d**)). (**e**) ICs in the serosal layer of the vas deferens. (**f**) Cell processes of ICs in the serosal layer. ICs’ cell bodies are relatively small, with sparse cytoplasm containing several mitochondria surrounding the nucleus (white arrows). ICs were in close proximity to each other (black arrows). Caveolae in the cytoplasm of ICs in all the layers (black arrowheads). IC, interstitial cells; SMC, smooth muscle cells; E, epithelium; MC, mesothelial cells. Scale bars: 2 μm (**a**–**e**); 500 nm (**f**, inset at the lower left corner of (**d**)).

We identified ICs in the lamina propria, smooth muscle layer, and serosal layer of murine vas deferens (Fig. 6a–f). ICs’ cell bodies are relatively small, with sparse cytoplasm containing several mitochondria surrounding the nucleus (Fig. 6a–f, white arrows). ICs in all layers were close proximity to each other (Fig. 6a–c,e, black arrows). These ICs had several elongated cellular processes. Within the cellular processes, dilated and thinned segments were observed. Organelles including mitochondria were observed in the dilated segments and the cytoplasm of these cells was not surrounded by a continuous basal lamina (Fig. 6b,d,f). A small number of caveolae were partially observed in the cytoplasm of ICs in all layers (Fig. 6b,d,f, black arrowheads). This was consistent with the immunolabeling results.

**Figure 7:**
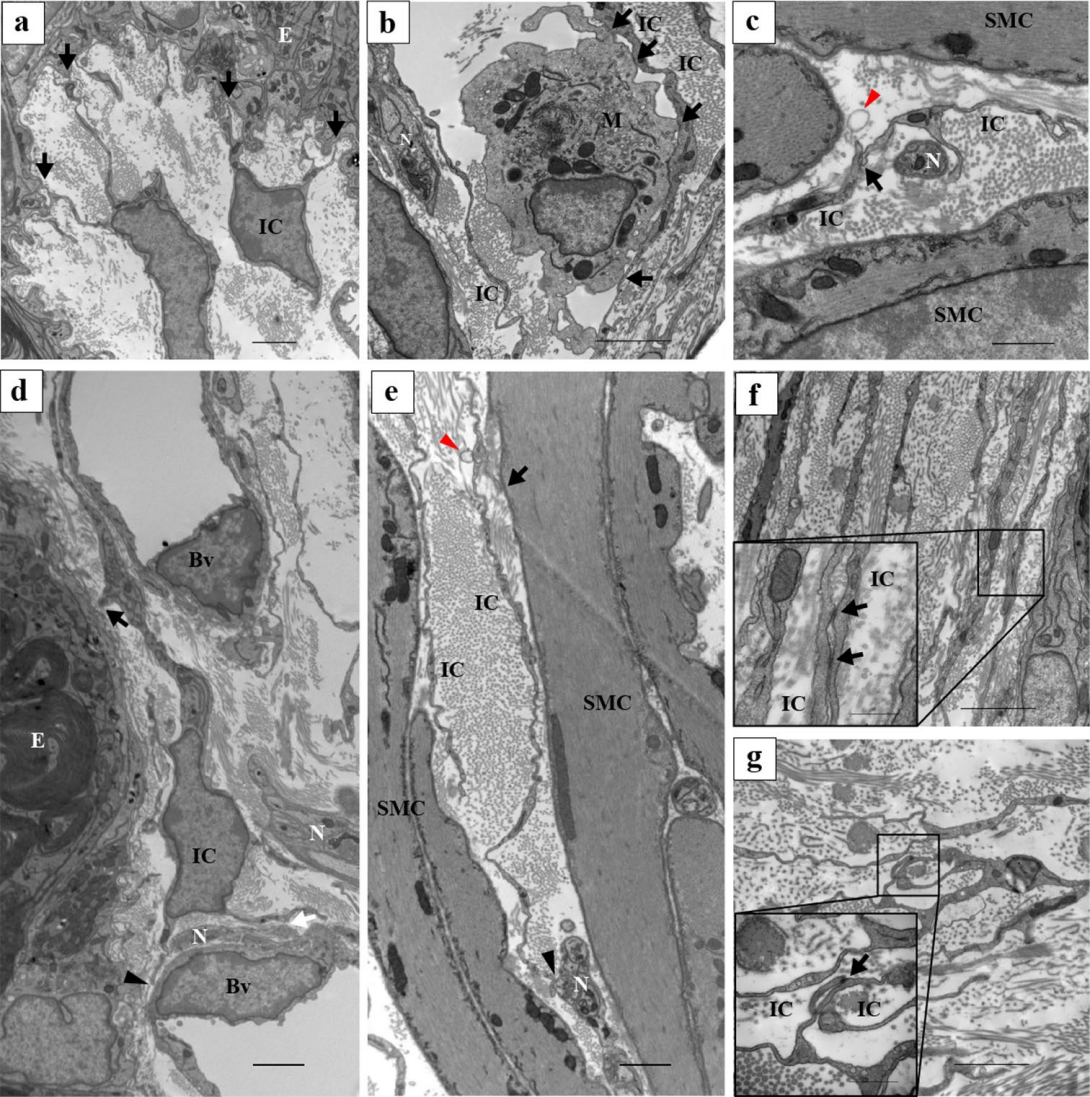
(**a**) The cell processes of the ICs were in close proximity to the epithelium (black arrows). (**b**) A cell process of the IC was close proximity to a macrophage (black arrows). A cell process of the IC wraps nerves and the close proximity to the cell process of the other IC (black arrows). (**c**) Exosome-like structure nearby cell process of the IC (red arrowhead). (**d**) The IC in the lamina propria were close proximity to surrounding tissues. The IC in the lamina propria was in close proximity to the epithelial cell (black arrow), a vascular endothelial cell (black arrowhead) and a nerve (white arrow). (**e**) The IC in the smooth muscle layer was in close proximity to the surrounding tissues. The IC in the smooth muscle layer was in close proximity to a smooth muscle cell (black arrow) and a nerve (black arrowhead). Exosome-like structure near the cell processes of IC (red arrowhead). (**f**,**g**) TEM images of contact areas between ICs. Higher magnification of the solid black line square area (inset at the lower left corner of (**f**,**g**)). Electron-dense lines exist in the contact area between ICs (black arrows). IC, interstitial cells; E, epithelium; N, nerves; Bv, blood vessels; M, macrophages. Scale bars: 2 μm (**a**–**g**); 500 µm (inset at the lower left corner of (**f**,**g**)).

ICs in all layers were in close proximity to not only each other but also the epithelium, nerves, vascular endothelial cells, and macrophages (Fig. 7a–c, black arrows), similar to the immunolabeling results. Moreover, the IC in the lamina propria was in close proximity to the epithelium (Fig. 7d, black arrow), a vascular endothelial cell (Fig. 7d, black arrowhead), and a nerve (Fig. 7d, white arrow). The IC in the smooth muscle layer was in close proximity to a smooth muscle cell (Fig. 7e, black arrow) and a nerve (Fig. 7e, black arrowhead).

Electron-dense lines were observed in the close proximity areas between the ICs (Fig. 7f,g, black arrows). These structures were not observed in close proximity areas between ICs and surrounding tissues, epithelial cells, smooth muscle cells and nerves. In addition, microvesicle-like structures or exosomes were frequently observed between ICs and surrounding tissues (Figs. 6d; 7c,e, red arrowheads).

### Ultrastructural identification of PDGFRα-IR using immuno-EM

**Figure 8:**
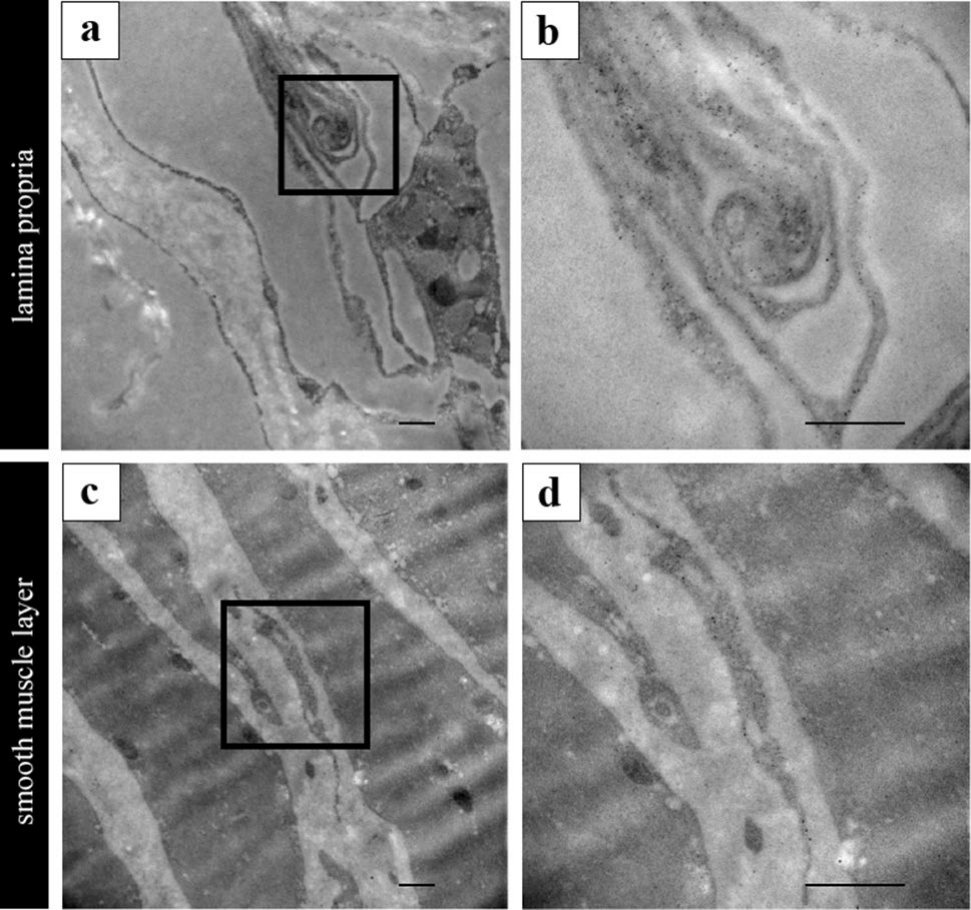
(**a**–**d**) Immunoelectron microscopy revealed ICs labeled PDGFRα in the lamina propria and smooth muscle layer. (**b**) High magnification of the solid black line square area in (**a**). (**d**) High magnification of the solid black square area in (**c**). A total of 10 nm colloidal golds homogeneously labeled in the cytoplasm of ICs (**b**,**d**). Scale bars: 2 μm (**a**,**c**); 500 nm (**b**,**d**).

Immuno-EM revealed that PDGFRα-IR correlated with ICs observed by TEM and confocal laser scanning microscopy with respect to their anatomical location, relative density, and approximate characteristics of the morphology. ICs of murine vas deferens were identified as PDGFRα-IR labeled with 10 nm colloidal golds (Fig. 8a,c); 10 nm colloidal golds homogeneously labeled most cytoplasm of ICs in the lamina propria and smooth muscle layer (Fig. 8b,d). This finding was the same as that of immunolabeling (Fig. 2d).

## Discussion

To the best of our knowledge, this is the first study to demonstrate the immunohistochemical, morphological, and ultrastructural characteristics of PDGFRα-positive ICs in mammalian vas deferens. As a preliminary step towards analyzing PDGFRα-ICs, we successfully obtained stereoscopic whole imaging data of murine vas deferens using a modified tissue clearing CUBIC method to determine the target segments. Since the vas deferens contains large amounts of myoglobin, we chose the CUBIC system that efficiently facilitates the decolorization of endogenous chromophores, such as myoglobin, within tissues^23^.

Our 3D reconstruction of the GFP transgenic murine vas deferens illustrated well-developed transverse folds towards the lumen in addition to the previously reported longitudinal folds^24,25^. The transverse folds appeared more frequent and were winding in a zigzag pattern to fill the luminal space and simultaneously rose towards the distal portion of the organ. The epithelium of the folds expanded and tightly occupied the luminal space, which might have been beneficial in enlarging the surface area to interact with contents, such as sperm and the surrounding fluid, for their metabolism, as well as the phagocytic activity of epithelial cells^26,27^. The structure of vas deferens changes greatly depending on the segment, and the contractions of the circular and longitudinal muscles of the vas deferens have different mechanisms^28^. For further investigation, we selected the distal segment, which had two types of muscularis as well as many complex structures.

Immunohistochemical analyses revealed that PDGFRα-positive ICs were present in the lamina propria, smooth muscle layer, and serosal layer of the murine vas deferens, and may have two different types of immunohistochemical characteristics in the lamina propria. CD34 has been widely used as a marker for labeling interstitial cells, such as PDGFRα and previous studies on other organs have reported that PDGFRα and CD34 are co-labeled frequently^29–31^, however, we found PDGFRα-single positive ICs beneath the epithelium and PDGFRα/CD34-double-positive ICs in the deeper portion. Vannucchi et al. reported that PDGFRα-positive ICs in the upper lamina propria of the human bladder were CD34 negative and PDGFRα positive-ICs in the deep lamina propria, whereas in the detrusor of the human bladder they were CD34-positive^32^. In addition, they reported that some of PDGFRα-positive ICs were also αSMA positive, referred to as ‘myoid’ ICs^32^, which are thought to be present in the interstitial space of organs in the urinary system, such as kidney and urinary bladder^32,33^. The PDGFRα/α-SMA double immunostaining did not reveal ‘myoid’ ICs in the murine vas deferens stromal compartment. It remains unknown whether the difference in immunochemical characteristics between PDGFRα-positive ICs beneath the epithelium and other PDGFRα-positive ICs is associated with functional characteristics, however, Shoshkes-Carmel et al. reported that PDGFRα-positive ICs beneath the epithelium in the gastrointestinal tract are an important source of niche signals to intestinal stem cells^34^. Functional significance of the differences in immunostaining patterns of the murine vas deferens, as seen in the gastrointestinal tract, warrants further investigation.

Previous studies in other organs have reported that PDGFRα and vimentin are co-labeled frequently^8^. In the present study, vimentin was labeled primarily in the cell body of PDGFRα-positive ICs and was partially labeled in the prolongations of PDGFRα-positive ICs. These findings indicate that the amount of intermediate filament, labeled by vimentin, is heterogeneous in the cell. Therefore, it is essential to distinguish between vimentin-positive or negative cells, based on the stained section, when conducting immunolabeling^35^.

The ICC of the gastrointestinal tracts express c-Kit. KIT, a receptor tyrosine-protein kinase, acts as a cell-surface receptor for the cytokine KIT ligand/stem cell factor (SCF) and plays an essential role in the regulation of cell survival and proliferation, differentiation, stem cell maintenance, and mast cell development^36,37^. In the present study, c-Kit-positive ICC were not observed. The presence of ICC networks in non-intestinal organs remains controversial. KIT is expressed in several cell types, such as mast cells, hematopoietic cells, spermatogonia, and melanocytic cells^38^. Of these populations, mast cells appear in most of the organs^39^; therefore, c-Kit-positive cells observed in this study were considered as mast cells based on their morphology. However, double staining for c-Kit and the mast cell tryptase is required for a definite identification of c-KIT-positive cells as mast cells.

We observed that PDGFRα-positive ICs in the murine vas deferens were not only in close proximity to each other but also with various cells and tissues including epithelial cells, nerves, vascular endothelial cells, macrophages, and smooth muscle cells, which were seen using immunofluorescence and TEM. According to previous reports, PDGFRα-positive ICs establish a 3D network of cell processes, and PDGFRα-positive ICs may be involved in nervous, vascular and immune systems, regulation of tissue homeostasis and long-distance communication through intercellular signaling due to their strategic position near other cells and tissues^40^. Our results were consistent with these findings, and PDGFRα-positive ICs in murine vas deferens may also be involved in intercellular signaling.

PDGFRα-positive ICs in the smooth muscle layers of the gastrointestinal tract express the SK3 channel, which plays a critical role in purinergic nerve-mediated smooth muscle relaxation^12,13^. SK3/PDGFRα-positive ICs have also been identified in the smooth muscle layers of bladder^15^ and seminal vesicle^20^. In addition, the ANO1 channel is expressed in PDGFRα-positive ICs of renal pelvis where it may influence their spontaneous activities^14^. In the present study, neither SK3 nor ANO-1 channels were detected in the PDGFRα-positive ICs of the smooth muscle layer of the murine vas deferens. The properties of antibodies used, animal species, and organs might have contributed to this.

A small number of caveolae were partially observed in the PDGFRα-positive ICs of murine vas deferens in the EM observations. Although the role of the caveolae of PDGFRα-positive ICs remains unclear, ICCs of the myenteric plexus have been reported to account for the recycling of calcium from caveolar domains to SR through Cav1 to sustain pacing and contractions^41^. In addition, gap junctions between PDGFRα-positive ICs may distribute more frequently in the lamina propria than in the smooth muscle layer depending on the distribution density of ICs. Previous studies have reported that PDGFRα-positive ICs exchange small molecules, so-called secondary molecular messengers with role in cellular signaling, via gap junctions^42,43^. No gap junctions were observed between PDGFRα-positive ICs and smooth muscle cells in the murine vas deferens. This finding supports the hypothesis that gap junctions expressed by PDGFRα-positive ICs are restricted to homocellular communications^44^. Connexin 43 does not definitively prove the existence of gap junctions^45^, and the observation of gap junctions with TEM is challenging since gap junctions may only be present in tiny contact areas between the PDGFRα-positive ICs. The 2–3 nm “gap” separating the plasma membranes is commonly observed in a high magnification view of the gap junction^43^. However, it is difficult to observe the “gap” in tiny contact areas between PDGFRα-positive ICs. Therefore, further investigations are warranted to elucidate the possible role of gap junctions in murine vas deferens.

We frequently identified fragmented vesicles, microvesicle-like structures or exosomes, between PDGFRα-positive ICs and surrounding tissues in our TEM images. It is possible that PDGFRα-positive ICs were supposed to transfer functional molecules to surrounding tissues and cells by exosomes. The term "stromal synapse" has been used to describe synaptic connections between PDGFRα-positive ICs and neighboring cells, speculated as a juxtracrine (a chemical synapse) and corresponding to an exosome-based mechanism^46^. The exosomes may act as specific containers filled with proteins, lipids, microRNA, and/or other materials that are transported to other neighboring stromal cells where they alter their function^47^. These vesicles transport RNA or DNA to target cells, inducing epigenetic changes^48^. Therefore, further research on exosome-like structures observed in the present study may demonstrate the function of PDGFRα-positive ICs.

Our study has three limitations. First, PDGFRα, as a cell marker, may also label various mesenchymal cells. Therefore, use of several antibodies can increase the immunohistochemical discrimination. Second, our findings and previous studies related to the morphological evaluation of PDGFRα-positive ICs have mostly used 2D images obtained from sections^18,29,30,49^. As PDGFRα-positive ICs are very elongated, and their cell processes multi-branched, the 3D evaluation would increase the accuracy of identifying such morphological changes. Visualization of GFP-positive cells in the 3D organ architecture by CUBIC method used in the present study is one of the choices to reveal 3D structure of PDGFRα-positive ICs. Finally, PDGFRα-positive ICs observed by CLSM are different from TEM observed cells. By a correlative light and electron microscopy (CLEM) method, more accurate ultrastructural information of PDGFRα-positive ICs can be obtained as the same cell samples are investigated using both light and electron microscopy.

In conclusion, our findings suggest that PDGFRα-positive ICs were located in the lamina propria, smooth muscle, and serosal layers of the murine vas deferens. In addition, PDGFRα-positive ICs may have several subpopulations depending on localization from immunohistochemical characteristics. Although we did not elucidate the presumptive functions of PDGFRα-positive ICs in murine vas deferens; they may however play an important role in intercellular signaling via direct contact of the IC network and extracellular vesicles, similar to previous reports on other organs. This study provides a novel structural and functional perspective on the cellular components of murine vas deferens. Our findings also progress our understanding of the physiological aspects of vas deferens. Further investigations on PDGFRα-positive ICs of the vas deferens may lead to understanding vas deferens motility.

## Supplementary Information

Supplementary Information

## Data Availability

All data generated or analyzed in this study are included in this article and the Supplementary Information Files.

## References

[1] Berridge MJ (2008). Smooth muscle cell calcium activation mechanisms. J. Physiol..

[2] Koslov DS, Andersson KE (2013). Physiological and pharmacological aspects of the vas deferens-an update. Front. Pharmacol..

[3] Hagger R, Finlayson C, Jeffrey I, Kumar D (1997). Role of the interstitial cells of Cajal in the control of gut motility. Br. J. Surg..

[4] Zhang RX, Wang XY, Chen D, Huizinga JD (2011). Role of interstitial cells of Cajal in the generation and modulation of motor activity induced by cholinergic neurotransmission in the stomach. Neurogastroenterol. Motil..

[5] Ward SM, Sanders KM, Hirst GD (2004). Role of interstitial cells of Cajal in neural control of gastrointestinal smooth muscles. Neurogastroenterol. Motil..

[6] Sanders KM (2006). Interstitial cells of Cajal at the clinical and scientific interface. J. Physiol..

[7] Komuro T (1999). Comparative morphology of interstitial cells of Cajal: Ultrastructural characterization. Microsc. Res. Tech..

[8] Aleksandrovych V (2017). Telocytes: Facts, speculations and myths (Review article). Folia. Med. Cracov..

[9] Andrae J, Gallini R, Betsholtz C (2008). Role of platelet-derived growth factors in physiology and medicine. Genes Dev.

[10] Edelstein L, Smythies J (2014). The role of telocytes in morphogenetic bioelectrical signaling: Once more unto the breach. Front. Mol. Neurosci..

[11] Mirancea N (2016). Telocyte—a particular cell phenotype. Infrastructure, relationships and putative functions. Rom. J. Morphol. Embryol..

[12] Baker SA, Hennig GW, Ward SM, Sanders KM (2015). Temporal sequence of activation of cells involved in purinergic neurotransmission in the colon. J. Physiol..

[13] Kurahashi M (2011). A functional role for the 'fibroblast-like cells' in gastrointestinal smooth muscles. J. Physiol..

[14] Grainger N (2020). Identification and classification of interstitial cells in the mouse renal pelvis. J. Physiol..

[15] Lee H, Koh BH, Peri LE, Sanders KM, Koh SD (2013). Functional expression of SK channels in murine detrusor PDGFR+ cells. J. Physiol..

[16] Yang C, Xiao J (2016). Editorial: Telocytes in regeneration and repair. Curr. Stem Cell Res. Ther..

[17] Varga I (2019). Recently discovered interstitial cell population of telocytes: Distinguishing facts from fiction regarding their role in the pathogenesis of diverse diseases called "Telocytopathies". Medicina.

[18] Liu Y (2019). Identification and characterization of telocytes in rat testis. Aging (Albany NY).

[19] Awad M, Ghanem ME (2018). Localization of telocytes in rabbits testis: Histological and immunohistochemical approach. Microsc. Res. Tech..

[20] Takeya M (2017). Role of mucosa in generating spontaneous activity in the guinea pig seminal vesicle. J. Physiol..

[21] Okabe M, Ikawa M, Kominami K, Nakanishi T, Nishimune Y (1997). 'Green mice' as a source of ubiquitous green cells. FEBS Lett..

[22] Walton J (1979). Lead aspartate, an en bloc contrast stain particularly useful for ultrastructural enzymology. J. Histochem. Cytochem..

[23] Susaki EA, Ueda HR (2016). Whole-body and whole-organ clearing and imaging techniques with single-cell resolution: Toward organism-level systems biology in mammals. Cell Chem. Biol..

[24] Kennedy SW, Heidger PM (1979). Fine structural studies of the rat vas deferens. Anat. Rec..

[25] Prins GS, Zaneveld LJ (1979). Distribution of spermatozoa in the rabbit vas deferens. Biol. Reprod..

[26] Murakami M, Nishida T, Iwanaga S, Shiromoto M (1984). Scanning and transmission electron microscopic evidence of epithelial phagocytosis of spermatozoa in the terminal region of the vas deferens of the cat. Experientia.

[27] Cooper TG, Hamilton DW (1977). Phagocytosis of spermatozoa in the terminal region and gland of the vas deferens of the rat. Am. J. Anat..

[28] Amobi NI, Guillebaud J, Smith IC (2012). Perspective on the role of P2X-purinoceptor activation in human vas deferens contractility. Exp. Physiol..

[29] Vannucchi MG, Traini C, Manetti M, Ibba-Manneschi L, Faussone-Pellegrini MS (2013). Telocytes express PDGFRα in the human gastrointestinal tract. J. Cell Mol. Med..

[30] Rosa I (2019). Telocytes constitute a widespread interstitial meshwork in the lamina propria and underlying striated muscle of human tongue. Sci. Rep..

[31] Rosa I, Marini M, Sgambati E, Ibba-Manneschi L, Manetti M (2020). Telocytes and lymphatic endothelial cells: Two immunophenotypically distinct and spatially close cell entities. Acta Histochem..

[32] Vannucchi MG, Traini C, Guasti D, Del Popolo G, Faussone-Pellegrini MS (2014). Telocytes subtypes in human urinary bladder. J. Cell Mol. Med..

[33] Desmoulière A, Darby IA, Gabbiani G (2003). Normal and pathologic soft tissue remodeling: Role of the myofibroblast, with special emphasis on liver and kidney fibrosis. Lab. Invest..

[34] Shoshkes-Carmel M (2018). Subepithelial telocytes are an important source of Wnts that supports intestinal crypts. Nature.

[35] Koh BH (2012). Platelet-derived growth factor receptor-α cells in mouse urinary bladder: A new class of interstitial cells. J. Cell Mol. Med..

[36] Kitamura Y, Hirotab S (2004). Kit as a human oncogenic tyrosine kinase. Cell Mol. Life Sci..

[37] Stankov K, Popovic S, Mikov M (2014). C-KIT signaling in cancer treatment. Curr. Pharm. Des..

[38] Huizinga JD, Chen JH (2014). Interstitial cells of Cajal: Update on basic and clinical science. Curr. Gastroenterol. Rep..

[39] Stone KD, Prussin C, Metcalfe DD (2010). IgE, mast cells, basophils, and eosinophils. J. Allergy Clin. Immunol..

[40] Gherghiceanu M, Popescu LM (2012). Cardiac telocytes—their junctions and functional implications. Cell Tissue Res..

[41] Daniel EE, Eteraf T, Sommer B, Cho WJ, Elyazbi A (2009). The role of caveolae and caveolin 1 in calcium handling in pacing and contraction of mouse intestine. J. Cell Mol. Med..

[42] Faussone-Pellegrini MS, Gherghiceanu M (2016). Telocyte's contacts. Semin. Cell Dev. Biol..

[43] Goodenough DA, Paul DL (2009). Gap junctions. Cold Spring Harb. Perspect. Biol..

[44] Popescu LM, Fertig ET, Gherghiceanu M (2016). Reaching out: Junctions between cardiac telocytes and cardiac stem cells in culture. J. Cell Mol. Med..

[45] Laird DW (2006). Life cycle of connexins in health and disease. Biochem. J..

[46] Popescu LM, Gherghiceanu M, Cretoiu D, Radu E (2005). The connective connection: Interstitial cells of Cajal (ICC) and ICC-like cells establish synapses with immunoreactive cells. Electron microscope study in situ. J. Cell Mol. Med..

[47] El-Tahawy NFG, Rifaai RA (2019). Immunohistochemical and ultrastructural evidence for telocytes in the different physiological stages of the female rat mammary gland. Life Sci..

[48] Yang P (2017). Cellular evidence of telocytes as novel interstitial cells within the magnum of chicken oviduct. Cell Transplant..

[49] Marini M (2017). Telocytes in normal and keratoconic human cornea: An immunohistochemical and transmission electron microscopy study. J. Cell Mol. Med..

