# Supplementary material for: Identification of PDGFRα-positive interstitial cells in the distal segment of the murine vas deferens
Source: Sci Rep. 2021 Apr 6;11:7553. doi: 10.1038/s41598-021-87049-6 (PMC8024294; doi:10.1038/s41598-021-87049-6)
Supplement: Supplementary file 1 — Supplementary Information [file 41598_2021_87049_MOESM1_ESM.pdf]

Identification of PDGFRα-positive interstitial cells in the distal segment of the murine vas deferens using light microscopy and transmission electron microscopy

Tasuku Hiroshige[1, 2]*, Kei-Ichiro Uemura[2], Shingo Hirashima[1], Kiyosato Hino[1], Akinobu Togo[3], Keisuke Ohta[1, 3], Tsukasa Igawa[2] and Kei-Ichiro Nakamura[1]

[1]Division of Microscopic and Development Anatomy, Department of Anatomy Kurume University School of Medicine, Kurume 830-0011, Japan; [2] Department of Urology Kurume University School of Medicine, Kurume 830-0011, Japan; [3] Advanced Imaging Research Center, Kurume University School of Medicine, Kurume 830-0011, Japan

***Corresponding author**: Tasuku Hiroshige, Division of Microscopic and Development Anatomy, Department of Anatomy Kurume University School of Medicine, Kurume 830-0011, Japan

E-mail: hiroshige_tasuku@med.kurume-u.ac.jp

Keywords: PDGFRα, interstitial cells, vas deferens

Running title: HIROSHIGE *et al*: PDGFRα-positive interstitial cells in murine vas deferens

Supplementary Table S1. Primary antibodies used in this study

**Primary antibody**	**Host**	**Working dilution**	**Source**
PDGFR-α	Goat	1:400	Catalog no. AF1062; R&D systems
Texas-red-phalloidin		1:100	Catalog no. T7471; Thermo fisher
CD34	Rat	1:900	Catalog no. 14-0341; eBioscience
Vimentin	Chicken	1:2,000	Catalog no. ab24525; abcam
c-Kit	Rat	1:250	Catalog no. ab112177; abcam
caveolin-1	Rabbit	1:500	Catalog no. ab2910; abcam
β3-tublin	Rabbit	1:500	Catalog no. ab18207; abcam
Iba-1	Rabbit	1:250	Catalog no. 019-19741; wako
αSMA	Rabbit	1:700	Catalog no. ab5694; abcam
αSMA	Goat	1:200	Catalog no. NB300-978; Novusbio
connexin43	Rabbit	1:1,000	Catalog no. ab34710; abcam
laminin	Rabbit	1:50	Catalog no. ab91006; abcam
SK3	Rabbit	1:500	A gift from Dr. Hayashi
ANO-1	Rabbit	1:500	Catalog no. ab53212; abcam

The specificity of the anti-SK3 antibody has been confirmed by Hayashi et al. (2019).

Supplementary Figure S1

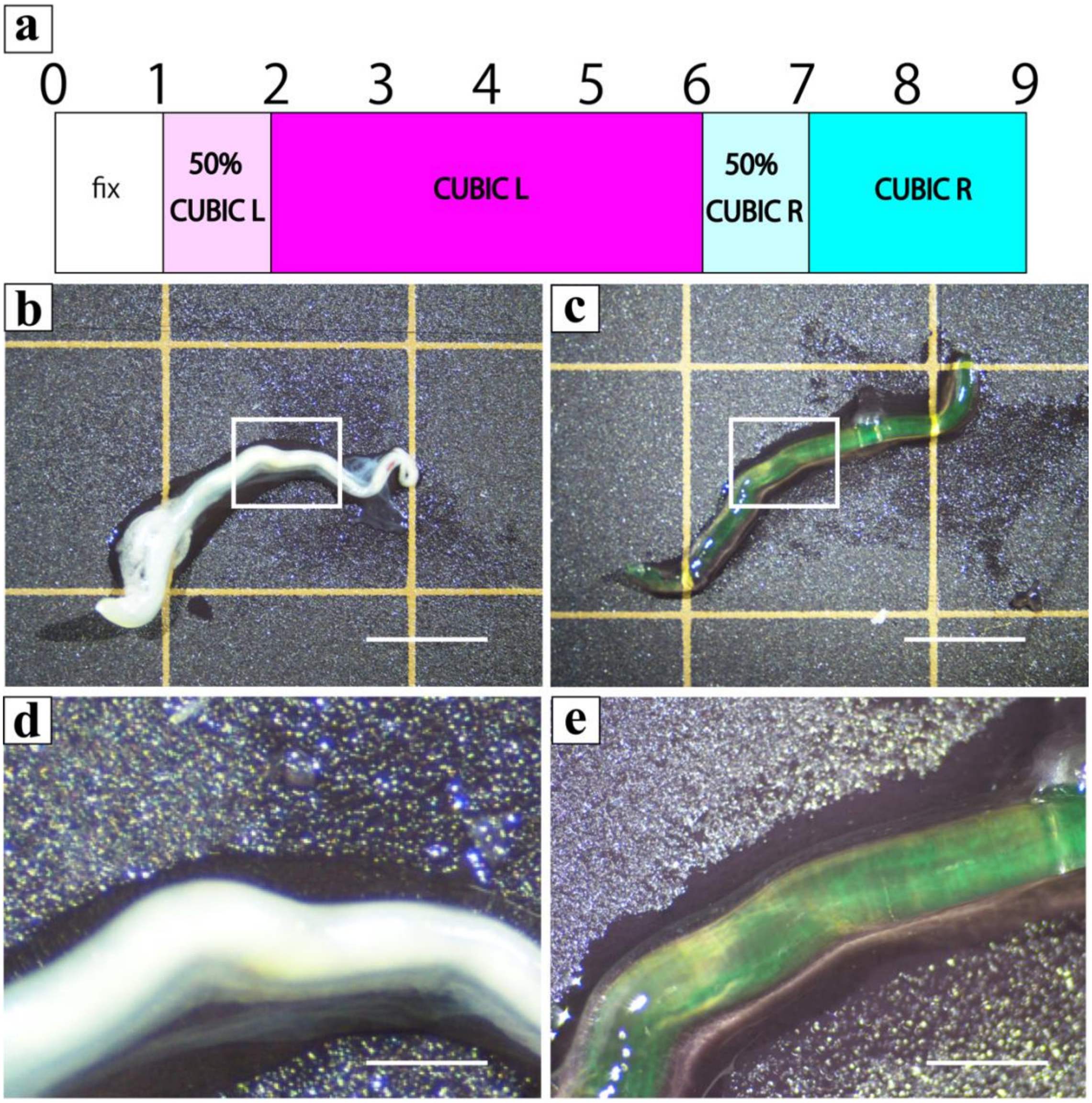

Tissue clearing of the GFP transgenic murine vas deferens was conducted using the CUBIC protocol (**a**). Bright-field images of the GFP transgenic murine vas deferens before (**b**) and after (**c**) tissue clearing (**b**, **c**). A highly magnified image of the white square of (**b**) (**d**). A highly magnified image of the white square of (**c**) (**e**). Scale bars: 5 mm (**b, c**); 1 mm (**d, e**).

Supplementary Figure S2

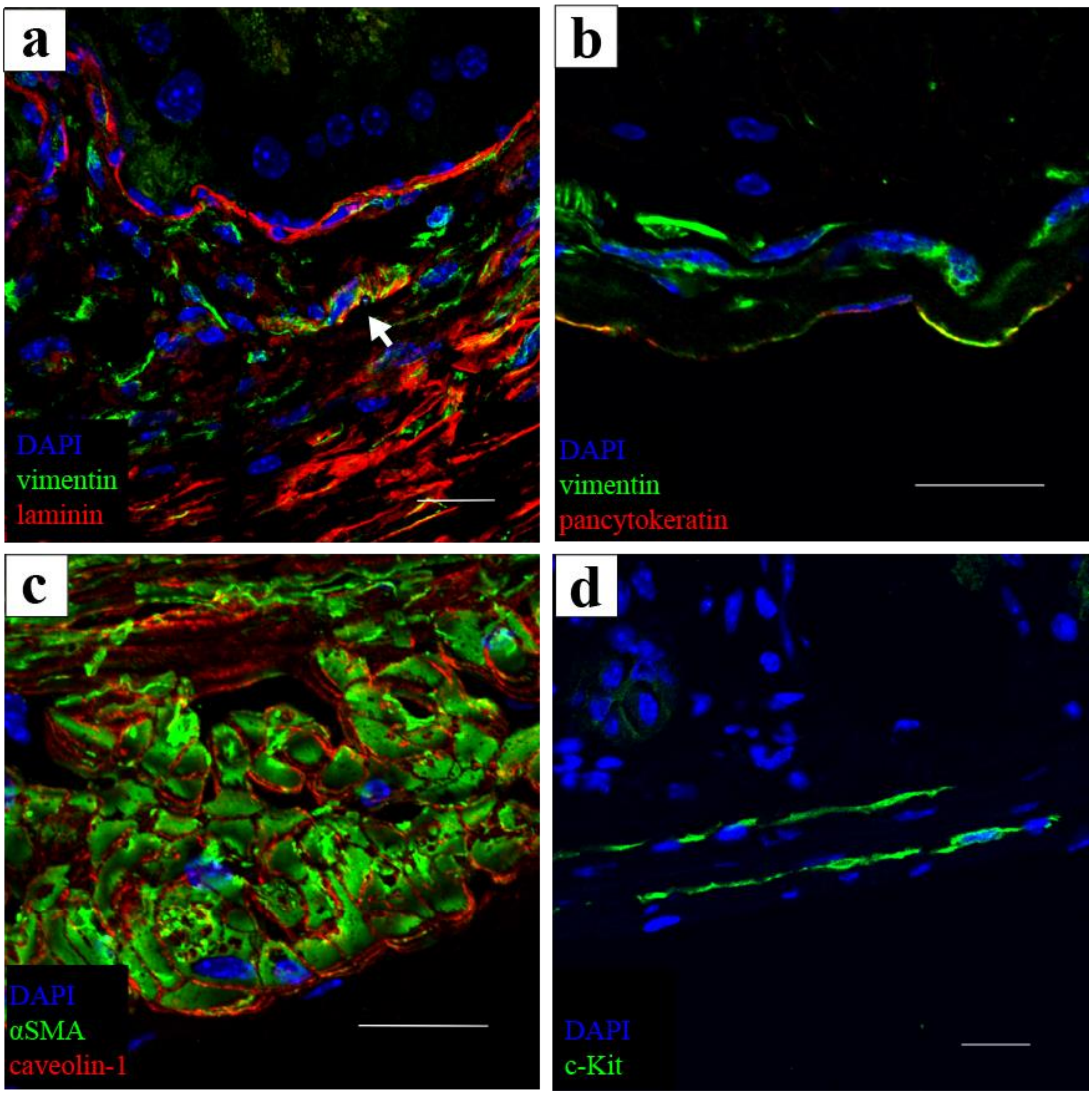

Representative images of double immune-labeling for vimentin (green) and laminin (red). Nuclei were counterstained in blue with DAPI (**a**). Vimentin-IR was partially co-labeled with laminin-IR (white arrow). Representative images of double immune-labeling for vimentin (green) and pan-cytokeratin (red). Nuclei were counterstained in blue with DAPI (**b**). Vimentin-IR observed in the outermost serosal layer was co-labeled with pan-cytokeratin-IR.

Representative images of double immune-labeling for α-SMA (green) and caveolin-1 (red). Nuclei were counterstained in blue with DAPI (**c**). Caveolin-1-IR was observed on the edge of α-SMA-IR. Representative images of immune-labeling for c-Kit (green). Nuclei were counterstained in blue with DAPI (**d**). C-Kit-immunoreactivity (IR) was observed in the tunica muscularis of the murine colon, which was used as a positive control.

Scale bar: 20 μm (**a**-**d**).
